# Evaluation of Laminated Composite Beam Theory Accuracy

**DOI:** 10.3390/ma15196941

**Published:** 2022-10-06

**Authors:** Yu-Ting Lyu, Tsung-Pin Hung, Her-Chang Ay, Hsiu-An Tsai, Yih-Cherng Chiang

**Affiliations:** 1 Department of Mold and Die Engineering, National Kaohsiung University of Science and Technology, Kaohsiung 807618, Taiwan; 2 Department of Mechanical Engineering, Cheng Shiu University, Kaohsiung 833301, Taiwan; 3 Center for Environmental Toxin and Emerging-Contaminant Research, Cheng Shiu University, Kaohsiung 833301, Taiwan; 4 Super Micro Mass Research & Technology Center, Cheng Shiu University, Kaohsiung 833301, Taiwan; 5 Metal Industries Research and Development Centre (MIRDC), Kaohsiung 811020, Taiwan; 6 Department of Mechanical Engineering, Chinese Culture University, Taipei 11114, Taiwan

**Keywords:** CFRP, UAV, laminated composite beam, finite element analysis, effective stiffness

## Abstract

Carbon fiber-reinforced polymer (CFRP) has been widely implemented in electric vehicle bodies and aircraft fuselage structures. The purpose of CFRP is to reduce the weight and impart rigidity in the final product. A beam structure is typically used to bear the structural load, and the rigidity of the beam can be changed by arranging the laminated fibers at different angles. In this study, a composite I-beam is used as an example in engineering components. Because the theoretical model of the superimposed composite I-beam is established, the theoretical formula is based on the theoretical assumptions of the two-dimensional composite beam, and is combined with the traditional composite plate theory to analyze the maximum bending stress, strain, and deflection. During the theoretical derivation, it is assumed that the flanges of the I-beams are divided into narrow and wide flanges. The beams are considered as structures of beams and flatbeds. When a narrow flange is loaded in the side, the wide flange has no lateral deformation, and the lateral moments are neglected. Therefore, the accuracy of this formula needs to be verified. The purpose of this study is to verify the accuracy of theoretical solutions for the deflection and stress analysis of composite beams. A finite element analysis model is used as the basis for comparing the theoretical solutions. The results indicate that when the aspect ratio of the beam is >15, the theoretical solution will have better accuracy. Without the addition of the material, when 0° ply is placed on the outermost layer of the flange of the nonsymmetric beam, the effective rigidity of the beam is increased by 4–5% compared with the symmetrical beam. The accuracy range of the theoretical solution for the composite beams can be accurately defined based on the results of this study.

## 1. Introduction

Isotropic materials are widely used and their theoretical basis, design specifications, and processing technology have matured. Single and homogeneous material properties have limited the development of materials. Research on composite materials has been developed towards a variety of applications; for example, Bistable morphing composites for energy-harvesting [1]. The composite structure can generate kinetic energy by means of piezoelectric actuation, thermal actuation, shape memory alloy actuation and magnetostrictive actuation. Venkata Siva C. Chillara [2] used shape memory alloys as fiber reinforced elastomers combined with thermosetting reinforced polymer laminates to make bistable laminate composites. Applications in biomedical magnesium alloy metal composites are also often discussed in the field of manufacturing and application [3,4]. Because of the stability of the magnesium alloy structure, it is often used in the biomedicine, aerospace, and automobile industries. However, it is a big problem in processing. B. Ratna Sunil [3] used the friction stir processing technique to fabricate surface metal matrix composites, and the properties of magnesium alloy composites are also discussed.

With the advancement of material technology and understanding of anisotropic materials, the application of high performance composite materials has gradually become common. Fiber-reinforced polymer composites have replaced traditional metal materials. Fiber-reinforced epoxy resin composites are also often used in the engineering field. Composite materials use polymer materials as the base material. The methods commonly used in the processing of materials, such as drilling, cutting or joining, are to make the material more convenient to use. Ammar H. Elsheikh [5] proposed an optimizer of the parasitism–predation algorithm (PPA), optimized for drilling the parameters of non-laminated glass fiber-reinforced epoxy composites (GFREC). In addition, he proposed an estimation method for the optimization of cutting parameters for basalt fiber-reinforced polymer composites [6]. This estimation model is composed of Long Short-Term Memory (LSTM) and Chimp Optimization Algorithm (CHOA). This method can successfully evaluate and optimize the quality characteristics of laser-cut composites. Ezzat A. Showaib [7] used vibration welding technology to join GFRP. The effect of fiber orientation on the strength of welded joints was investigated for the joined materials.

The main reason for this is that composite materials have the advantages of light weight, high specific strength, and high specific rigidity. Owing to these advantages, several laminated composite materials have been used in the construction, machinery, and aerospace industries. However, the mechanical properties of the composite materials, selection of the materials, and optimal design of the structure are important topics in current studies. The use of composite materials in aircraft construction began in the 1940s with glass fiber-reinforced polymer as the backbone. Boron and carbon fibers were developed in the 1960s, and aramid fibers, developed in the 1970s, have also been successfully used in aviation structures. Carbon fiber-reinforced polymer (CFRP) has become the backbone of composite materials owing to its advantages such as low cost and ease of processing. The F-16 fighter fin outer panel is an example of CFRP application. With the gradual increase in demand for CFRP applications, the waste of related composite materials has also increased. It is estimated that the European market will reach 304,000 tons of thermosetting glass fiber composite waste per year, and the US CFRP waste is estimated to be approaching 2 million tons. Therefore, the treatment mechanism of composite material waste is becoming an increasingly important issue. Currently, composite materials can be reused by cutting and crushing waste composite materials. The recycled composite material parts are then pulverized to form a composite material powder. Thermosetting composite powders are often found in fillers. For example, thermosetting composite powder is often added to cement to increase its strength.

Unmanned aerial vehicles (UAVs) are playing an increasingly important role in the aviation industry. This is particularly true in military applications. CFRP helps fuselage structures reduce their weight by replacing current aerospace aluminum alloy structures, which improves the UAV airtime by reducing fuel consumption. Commercially, owing to the reduced weight of the fuselage structure, they can carry higher-capacity batteries and heavier loads. UAVs must ensure high-quality flight safety during navigation. This requires an accurate assessment of the structural integrity of the airframe. To achieve this, it is necessary to gain insight into the force modes of these advanced composite materials, as well as to assess the strength of the composite aerostructures. Alvarez-Montoya [8] proposed the use of optical fiber sensors to obtain the strain signal of a wing composite structure, which can monitor the health of the structure during flight. Gadomski [9] used finite element analysis to analyze the stiffness, stress, and vertical displacement distribution of carbon–epoxide composite beams in a wing and optimize the structure. Numerical scores are also used for predictions in the analysis of composite wing structures [10]. Alsahlani proposed the use of a low-order composite structure module to calculate typical wing structures. Finite-element software was used to verify the results. The results indicated that the maximum shear stress error was <4%. Galatas [11] combined the advantages of fused deposition modelling to rapidly produce complex shapes with composite sandwich structures. The mechanical properties of CFRP were investigated using an artificial neural network, which was used to improve the structural strength of the fixture of a quadrotor UAV, excluding the beam structure. Zhang [12] proposed an optimal design method to evaluate the stress and deflection of sandwich box girders composed of carbon fiber and polymethacryimide foam. This optimized method can be used to design lightweight spars for solar-powered UAVs.

Composite materials are used as structural components in several building materials. Abdulhameed [13,14,15] reported techniques for fabricating short-span beams from CFRP in the form of wedge-shaped concrete segments and laminates. This composite beam is primarily used for large-scale segmental beams in the construction of bridges. In addition, a new off-site and self-form segmental concrete masonry arch fabrication technique was proposed. Wedge-shaped plain concrete voussoirs and CFRP composites are used to manufacture arches. The CFRP improved the voussoir sliding load by 107%.

Beams are important structural components. The basic assumption of the theoretical analysis is that for a slender isotropic beam, after the cantilever beam or simple supported beam is subjected to bending deformation, the section is still perpendicular to the neutral plane without deformation, and the deformation caused by shear force is ignored. The deformation and displacement of warping, twisting, and extension caused by beam stress are ignored. These assumptions were also used for the analysis of heterogeneous beams. For the analysis of composite beams, Bank [16,17,18] modified the beam theory. The beam deformation is based on the assumptions of the Timoshenko [19] and Euler–Bernoulli beam theories, considering heterogeneity. The beam was subjected to three types of deformation: (1) deflection caused by bending when subjected to a transverse load or pure bending load; (2) rotation caused by twisting; and (3) displacement caused by axial extension. Therefore, the prediction of the deformation of the composite beam was more accurate. Yang [20] used the plate theory to derive the effect of shear deformation. Chandra [21] conducted theoretical and experimental static analyses of a laminated I-type composite beam. Based on the Vlasov type linear theory [22], the beam was subjected to tip bending and torsional load, and considering the influence of the beam’s lateral shear deformation, the deformation of the beam was predicted and compared with the experimental data.

Similar studies were conducted by Maddur and Chaturvedi. The Vlasov type [22] was used to modify the first-order shear deformation theory to analyze the dynamic response of an open-section composite beam. In addition, other numerical methods have been used to solve the approximate values. Minguet and Dugundji et al. used a finite difference solution in conjunction with experiments to analyze the static behavior of composite blades when they are subjected to significant deflections. According to Rehfield and Atilgan [23], the assumption for thin-walled beams is that only the deformation caused by the lateral shear force is considered in the buckling analysis of open-section composite materials. Stemple [24] used the finite element method (FEM) to analyze the effect of warping on composite beams. Gordaninejad [25] used a combination of two different materials, graphite/epoxy and aramid/rubber, to analyze the displacement of the neutral plane of the beam after bending, considering the deformation caused by the shear force.

In addition to the traditional composite theoretical analysis, Madenci [26,27] used mixed FEM equations and proposed a higher-order shear deformation theory to analyze the shear stress of the laminated composite. A bending analysis of the functionally graded material beams was also performed. This was combined with an artificial neural network algorithm to estimate the maximum displacement of a functionally graded material beam. Reddy [28] established a variable displacement FEM model for equivalent single-layer and layerwise laminate theories. This analytical model can be used to calculate the stresses in local areas of laminated composite structures.

Previous studies have mainly focused on discussing the external forces of composite beams, such as bending, torsion, extension loads, and beam deformation (warping, deflection, torsion, and extension) predictions. Theories were proposed and experiments were conducted to verify this. Hence, under the basic assumptions of the beam and laminated plate theories [29,30], both short beams (L/h < 10) and long beams (L/h > 10) ignore shear, torsion, warping, bending, and elongation. The aim was to simplify the formula and calculation. Several simplifying assumptions were made during the derivation of the mathematical model; however, the accuracy of the derivation formula was not discussed. Therefore, the main purpose of this study is to verify the accuracy of the formula through FEM analysis, with the aim of providing a reference for future designers.

## 2. Theory of Composite Beams

### 2.1. Symmetrical Laminated Composite I-Beam

**Figure 1:**
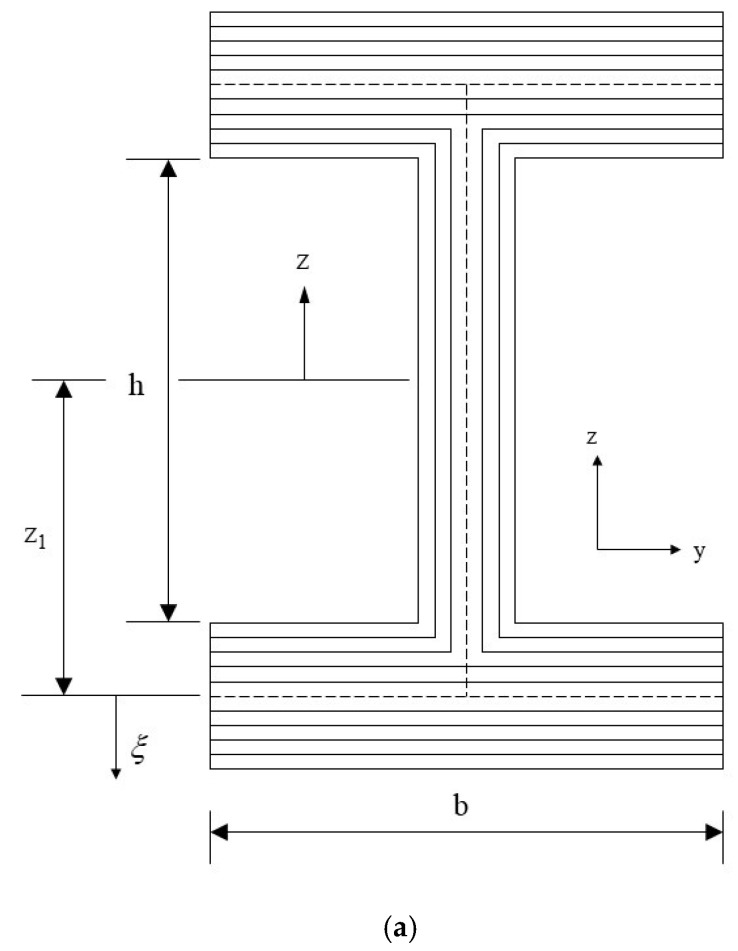

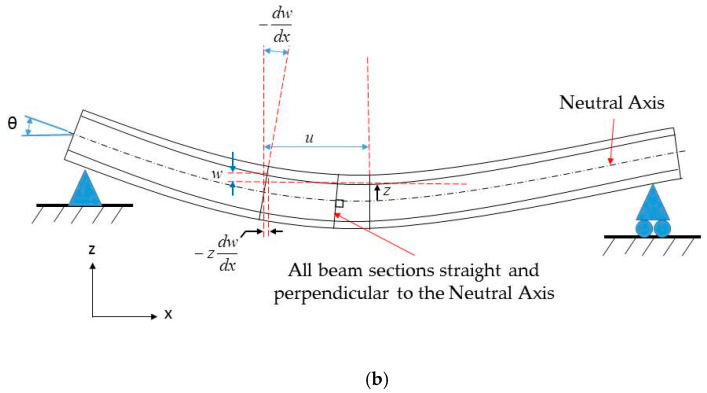
Laminated composite I-beam. (**a**) Cross section of laminated composite I-beam, and (**b**) I-beam theory assumptions [22].

The laminated I-beam shown in Figure 1a can be divided into a combination of three laminated plates of the web and upper and lower flanges. The assumptions of the composite beam based on the Euler–Bernoulli beam theory are as follows. (1) The deflection of the beam is smaller than the length of the beam (Figure 1b). Therefore, the deformed angles (θ) of the beam are small and the square of the slope is significantly smaller and can be ignored. (2) The section originally perpendicular to the central axis of the beam remained flat after bending, and was perpendicular to the central axis after bending. The displacement field can be expressed as [22]:

$$u_{1}(x,z)=u-z\frac{dw}{dx},\;u_{1}=0,\;u_{3}=w\left(x\right)$$

During analysis, the flange was divided into two hypotheses: narrow and wide flanges. The assumption of a narrow flange is that the beam width is approximately equal to the beam height; therefore, it can be considered as a beam. When the beam is subjected to a lateral load, the lateral strain is generated, and the lateral moment can be ignored. The assumption of a wide flange is that the beam width is significantly larger than the beam height. The beam could be regarded as a thin plate. Because of the lateral load, there is a lateral moment. In-plane stress analysis of the web and flange is discussed in detail below.

**Figure 2:**
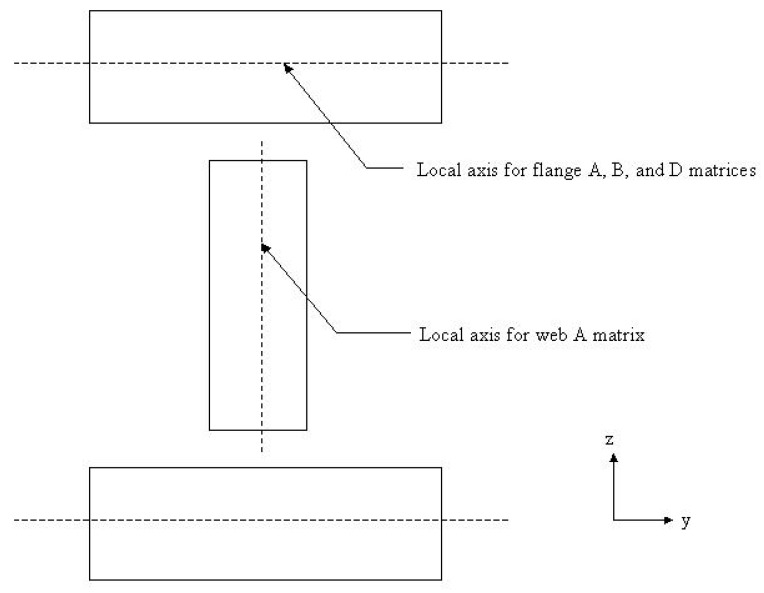
Matrix distribution of web and flange of I-beam [22].

The laminations in the web are parallel to the z-axis (Figure 2). The direction of the applied external force is not perpendicular to the laminations in the web; therefore, it is unrelated to the D matrix. From the stress–strain relations in Equation (1), the relationship between the normal stress and strain at the neutral axis is given by Equation (2):

(1) $$\left\{\begin{array}{c} \varepsilon_{x}^{0}\\ \varepsilon_{y}^{0}\\ \gamma_{xy}^{0} \end{array}\right\}=\left[A^{-1}\right]\left\{\begin{array}{c} N_{x}\\ 0\\ 0 \end{array}\right\}$$

(2) $$\varepsilon_{x}^{0}=A_{11\left(web\right)}^{-1}N_{x}$$

The axial strain of the web on the neutral axis is then:

(3) $$\varepsilon_{x}^{0}=z\kappa_{x}$$

From Equations (2) and (3), the normal stress on the web is:

(4) $$N_{x\left(web\right)}=\left[\frac{1}{A_{11\left(web\right)}^{-1}}\right]z\kappa_{x}$$

The relation between the moment and stress on the web can be obtained from the neutral axis of the I-beam as the origin and integral of the moment arm. The moment on the web can be written as:

(5) $$M_{web}=\int_{\frac{-h}{2}}^{\frac{h}{2}}N_{x}zdz=\int_{\frac{-h}{2}}^{\frac{h}{2}}\left[\frac{1}{A_{11\left(web\right)}^{-1}}\right]z^{2}\kappa_{x}dz=\frac{h^{3}\kappa_{x}}{12A_{11(web)}^{-1}}$$

- Narrow flange

Under a transverse load, the transverse resultant force, *N_y_* = 0, and bending moments, $M_{y}=0$ and $M_{xy}=0$, the relation between the strain, resultant force, curvature, and the resultant moment of the narrow-flange I-beam are as follows:

(6) $$\left\{\begin{array}{c} \varepsilon_{x}^{0}\\ \varepsilon_{y}^{0}\\ \gamma_{xy}^{0} \end{array}\right\}=\left[A^{-1}\right]\left\{\begin{array}{c} N_{x}\\ 0\\ 0 \end{array}\right\},\;\left\{\begin{array}{c} \kappa_{x}\\ \kappa_{y}\\ \kappa_{xy} \end{array}\right\}=\left[D^{-1}\right]\left\{\begin{array}{c} M_{x}\\ 0\\ 0 \end{array}\right\}$$

Equation (6) may be simplified to Equation (7):

(7) $$N_{x}=\frac{1}{A_{11,flange}^{-1}}\varepsilon_{x}^{0}=\frac{Z_{1}}{A_{11,flange}^{-1}}\kappa_{x},\;\mathrm{and}\;M_{x}=\frac{1}{D_{11,flange}^{-1}}\kappa_{x}$$

The moment of the flange is calculated from Equation (7) as follows:

(8) $$M_{flange}=b_{f}\left(N_{x}Z_{1}+M_{x}\right)=b_{f}\left(\frac{Z_{1}^{2}}{A_{11,flange}^{-1}}+\frac{1}{D_{11,flange}^{-1}}\right)\kappa_{x}$$

The moment of the beam is the sum of the flange and web moments:

(9) $$M_{beam}=M_{web}+2M_{flange}=EI_{eff}\kappa_{x}$$

Expanding Equation (9), the effective stiffness of the beam can be obtained:

(10) $$EI_{eff}=\frac{h^{3}}{12A_{11,web}^{-1}}+2b_{f}\left(\frac{Z_{1}^{2}}{A_{11,flange}^{-1}}+\frac{1}{D_{11,flange}^{-1}}\right)$$

The beam strain in the *x* direction is:

(11) $$\varepsilon_{x}=z\kappa_{x}$$

The relations between strain and curvature give:

(12) $$\left\{\begin{array}{c} \varepsilon_{x}\\ \varepsilon_{y}\\ \gamma_{xy} \end{array}\right\}=\left\{\begin{array}{c} \varepsilon_{x}^{0}\\ \varepsilon_{y}^{0}\\ \gamma_{xy}^{0} \end{array}\right\}+\xi \left\{\begin{array}{c} \kappa_{x}\\ \kappa_{y}\\ \kappa_{xy} \end{array}\right\}$$

From Equations (6) and (12), the strain in each ply of the flange is expressed as:

(13) $$\left\{\begin{array}{c} \varepsilon_{x}\\ \varepsilon_{y}\\ \gamma_{xy} \end{array}\right\}=\left\{\begin{array}{c} Z_{1}+\xi \\ \frac{A_{21,f}^{-1}}{A_{11,f}^{-1}}Z_{1}+\xi \frac{D_{21,f}^{-1}}{D_{11,f}^{-1}}\\ \frac{A_{31,f}^{-1}}{A_{11,f}^{-1}}Z_{1}+\xi \frac{D_{31,f}^{-1}}{D_{11,f}^{-1}} \end{array}\right\}\kappa_{x}$$

The stress of each ply of the flange can be obtained from the stress–strain relationship shown in Equation (14):

(14) $$\left\{\begin{array}{c} \sigma_{x}^{}\\ \sigma_{y}^{}\\ \tau_{xy}^{} \end{array}\right\}=\left[\begin{array}{ccc} {\bar{Q}}_{11} & {\bar{Q}}_{12} & {\bar{Q}}_{16}\\ {\bar{Q}}_{21} & {\bar{Q}}_{22} & {\bar{Q}}_{26}\\ {\bar{Q}}_{61} & {\bar{Q}}_{62} & {\bar{Q}}_{66} \end{array}\right]\left\{\begin{array}{c} \varepsilon_{x}^{}\\ \varepsilon_{y}^{}\\ \gamma_{xy}^{} \end{array}\right\}$$

- Wide flange

Under the assumption of a wide flange, the transverse resultant force and transverse curvature are zero, and the relationship between the strain, resultant force and curvature, and resultant moment of the I-beam with a wide flange becomes:

(15) $$\left\{\begin{array}{c} \varepsilon_{x}^{0}\\ \varepsilon_{y}^{0}\\ \varepsilon_{xy}^{0} \end{array}\right\}=\left[A^{-1}\right]\left\{\begin{array}{c} N_{x}\\ 0\\ 0 \end{array}\right\},\;\left\{\begin{array}{c} M_{x}\\ M_{y}\\ M_{xy} \end{array}\right\}=\left[D\right]\left\{\begin{array}{c} \kappa_{x}\\ 0\\ 0 \end{array}\right\}$$

Simplifying Equation (15), the resultant force and moment can be written as:

(16) $$N_{x}=\frac{1}{A_{11,flange}^{-1}}\varepsilon_{x}^{0}=\frac{Z_{1}}{A_{11,flange}^{-1}}\kappa_{x},\;\mathrm{and}\;M_{x}=D_{11,flange}\kappa_{x}$$

The moment of the flange is then calculated from Equation (16) as follows:

(17) $$M_{flange}=b_{f}\left(N_{x}Z_{1}+M_{x}\right)=b_{f}\left(\frac{Z_{1}^{2}}{A_{11,flange}^{-1}}+D_{11,flange}\right)\kappa_{x}$$

The effective stiffness of wide flange beams is derived from Equation (9):

(18) $$EI_{eff}=\frac{h^{3}}{12A_{11,web}^{-1}}+2b_{f}\left(\frac{Z_{1}^{2}}{A_{11,flange}^{-1}}+D_{11,flange}\right)$$

The strain of the beam in the *x* direction is shown in Equation (11).

From Equations (12) and (15), the strain of each ply of the flange is deduced as follows:

(19) $$\left\{\begin{array}{c} \varepsilon_{x}\\ \varepsilon_{y}\\ \gamma_{xy} \end{array}\right\}=\left\{\begin{array}{c} Z_{1}+\xi \\ \frac{A_{21}^{-1}}{A_{11}^{-1}}Z_{1}\\ \frac{A_{31}^{-1}}{A_{11}^{-1}}Z_{1} \end{array}\right\}\kappa_{x}$$

The stress derivation is as shown in Equation (14).

### 2.2. Nonsymmetrical Laminated Composite I-Beam

- Narrow flange

There is a B matrix in the nonsymmetric matrix; therefore, the F matrix is used to represent the inverse matrix of the ABD matrix:

(20) $$\left[F\right]\equiv {\left[\begin{array}{cc} A & B\\ B & D \end{array}\right]}^{-1}$$

Assuming that only the axial resultant force *N_x_* and the resultant bending moment *M_x_* exist, the relations among the strain, curvature, resultant force, and resultant moment of the nonsymmetric narrow flange I-beam are as follows:

(21) $$\left\{\begin{array}{c} \varepsilon_{x}^{0}\\ \varepsilon_{y}^{0}\\ \gamma_{xy}^{0}\\ \kappa_{x}\\ \kappa_{y}\\ \kappa_{xy} \end{array}\right\}=\left[\begin{array}{cccccc} F_{11} & F_{12} & F_{12} & F_{14} & F_{15} & F_{16}\\ F_{21} & F_{22} & F_{23} & F_{24} & F_{25} & F_{26}\\ F_{31} & F_{32} & F_{33} & F_{34} & F_{35} & F_{36}\\ F_{41} & F_{42} & F_{43} & F_{44} & F_{45} & F_{46}\\ F_{51} & F_{52} & F_{53} & F_{54} & F_{55} & F_{56}\\ F_{61} & F_{62} & F_{63} & F_{64} & F_{65} & F_{66} \end{array}\right]\left\{\begin{array}{c} N_{x}\\ 0\\ 0\\ M_{x}\\ 0\\ 0 \end{array}\right\}$$

Thus, $N_{x}$ and $M_{x}$ can be obtained as follows:

(22) $$N_{x}=\frac{F_{44}}{\Delta }\varepsilon_{x}^{0}-\frac{F_{14}}{\Delta }\kappa_{x},\;M_{x}=\frac{-F_{41}}{\Delta }\varepsilon_{x}^{0}+\frac{F_{11}}{\Delta }\kappa_{x}$$

where $\Delta \equiv F_{11}F_{44}-F_{14}F_{41}$

From Equation (9), the effective stiffness of the beam can be derived as follows:

(23) $$EI_{eff}=\frac{h^{3}}{12A_{11,web}^{-1}}+2b_{f}\left[\left(\frac{F_{44}Z_{1}}{\Delta }-\frac{F_{14}}{\Delta }\right)Z_{1}+\left(\frac{-F_{41}Z_{1}}{\Delta }+\frac{F_{11}}{\Delta }\right)\right]$$

From Equations (12), (21) and (22), the strain of each ply of the flange is derived as follows:

(24) $$\left\{\begin{array}{c} \varepsilon_{x}\\ \varepsilon_{y}\\ \gamma_{xy} \end{array}\right\}=\left[\begin{array}{cc} F_{11} & F_{14}\\ F_{21} & F_{24}\\ F_{31} & F_{34} \end{array}\right]\left[\begin{array}{c} N_{x}\\ M_{x} \end{array}\right]+\xi \left\{\begin{array}{c} \kappa_{x}\\ F_{51}N_{x}+F_{54}M_{x}\\ F_{61}N_{x}+F_{64}M_{x} \end{array}\right\}$$

The stress can then be obtained from Equation (14).

- Wide flange

The assumption of the nonsymmetric wide flange is that the transverse resultant force, $N_{y}=0$, and transverse curvature, $\kappa_{y}$ = 0, and the relation between the strain, curvature, resultant force, and resultant moment of the I-beam is as follows:

(25) $$\left\{\begin{array}{c} N_{x}\\ 0\\ 0\\ M_{x}\\ M_{y}\\ M_{xy} \end{array}\right\}=\left[\begin{array}{cccccc} A_{11} & A_{12} & A_{13} & B_{11} & B_{12} & B_{13}\\ A_{21} & A_{22} & A_{23} & B_{21} & B_{22} & B_{23}\\ A_{31} & A_{32} & A_{33} & B_{31} & B_{32} & B_{33}\\ B_{11} & B_{12} & B_{13} & D_{11} & D_{12} & D_{13}\\ B_{21} & B_{22} & B_{23} & D_{21} & D_{22} & D_{23}\\ B_{31} & B_{32} & B_{33} & D_{31} & D_{32} & D_{33} \end{array}\right]\left\{\begin{array}{c} \varepsilon_{x}^{0}\\ \varepsilon_{y}^{0}\\ \gamma_{xy}\\ \kappa_{x}\\ 0\\ 0 \end{array}\right\}$$

The inverse of the ABD matrix represented by the *F* matrix is given by Equation (26):

(26) $$\left\{\begin{array}{c} \varepsilon_{x}^{0}\\ \varepsilon_{y}^{0}\\ \gamma_{xy}^{0}\\ \kappa_{x}\\ 0\\ 0 \end{array}\right\}=\left[\begin{array}{cccccc} F_{11} & F_{12} & F_{12} & F_{14} & F_{15} & F_{16}\\ F_{21} & F_{22} & F_{23} & F_{24} & F_{25} & F_{26}\\ F_{31} & F_{32} & F_{33} & F_{34} & F_{35} & F_{36}\\ F_{41} & F_{42} & F_{43} & F_{44} & F_{45} & F_{46}\\ F_{51} & F_{52} & F_{53} & F_{54} & F_{55} & F_{56}\\ F_{61} & F_{62} & F_{63} & F_{64} & F_{65} & F_{66} \end{array}\right]\left\{\begin{array}{c} N_{x}\\ 0\\ 0\\ M_{x}\\ M_{y}\\ M_{xy} \end{array}\right\}$$

Simplifying Equation (26) to Equation (27), the new matrix is the *G* matrix:

(27) $$\left\{\begin{array}{c} \varepsilon_{x}^{0}\\ \kappa_{x}^{}\\ 0\\ 0 \end{array}\right\}=\left[\begin{array}{cccc} F_{11} & F_{14} & F_{15} & F_{16}\\ F_{41} & F_{44} & F_{45} & F_{46}\\ F_{51} & F_{54} & F_{55} & F_{56}\\ F_{61} & F_{64} & F_{65} & F_{66} \end{array}\right]\left\{\begin{array}{c} N_{x}\\ M_{x}\\ M_{y}\\ M_{xy} \end{array}\right\}=\left[\begin{array}{cccc} G_{11} & G_{12} & G_{13} & G_{14}\\ G_{21} & G_{22} & G_{23} & G_{24}\\ G_{31} & G_{32} & G_{33} & G_{34}\\ G_{41} & G_{42} & G_{43} & G_{44} \end{array}\right]\left\{\begin{array}{c} N_{x}\\ M_{x}\\ M_{y}\\ M_{xy} \end{array}\right\}$$

Then,

(28) $$\left\{\begin{array}{c} N_{x}\\ M_{x}\\ M_{y}\\ M_{xy} \end{array}\right\}={\left[\begin{array}{cccc} G_{11} & G_{12} & G_{13} & G_{14}\\ G_{21} & G_{22} & G_{23} & G_{24}\\ G_{31} & G_{32} & G_{33} & G_{34}\\ G_{41} & G_{42} & G_{43} & G_{44} \end{array}\right]}^{-1}\left\{\begin{array}{c} \varepsilon_{x}^{0}\\ \kappa_{x}^{}\\ 0\\ 0 \end{array}\right\}$$

Expanding Equation (28), the relationship between the stress, moment, and curvature of the flange is given by Equations (29)–(32):

(29) $$N_{x}=G_{11}^{-1}\varepsilon_{x}^{0}+G_{12}^{-1}\kappa_{x}=\left(Z_{1}G_{11}^{-1}+G_{12}^{-1}\right)\kappa_{x}$$

(30) $$M_{x}=G_{21}^{-1}\varepsilon_{x}^{0}+G_{22}^{-1}\kappa_{x}=\left(Z_{1}G_{21}^{-1}+G_{22}^{-1}\right)\kappa_{x}$$

(31) $$M_{y}=G_{31}^{-1}\varepsilon_{x}^{0}+G_{32}^{-1}\kappa_{x}=\left(Z_{1}G_{31}^{-1}+G_{32}^{-1}\right)\kappa_{x}$$

(32) $$M_{xy}=G_{41}^{-1}\varepsilon_{x}^{0}+G_{42}^{-1}\kappa_{x}=\left(Z_{1}G_{41}^{-1}+G_{42}^{-1}\right)\kappa_{x}$$

From Equations (9) and (28), the effective stiffness of the beam can be derived as follows:

(33) $$EI_{eff}=\frac{h^{3}}{12A_{11,web}^{-1}}+2b_{f}\left[G_{11}^{-1}Z_{1}^{2}+\left(G_{12}^{-1}+G_{21}^{-1}\right)Z_{1}+G_{22}^{-1}\right]$$

The strains for each ply of the flanges were obtained using Equations (12), (26) and (28). The formula is as follows:

(34) $$\left\{\begin{array}{c} \varepsilon_{x}\\ \varepsilon_{y}\\ \gamma_{xy} \end{array}\right\}=\left\{\begin{array}{cccc} F_{11} & F_{14} & F_{15} & F_{16}\\ F_{21} & F_{24} & F_{25} & F_{26}\\ F_{31} & F_{34} & F_{35} & F_{36} \end{array}\right\}\left\{\begin{array}{c} Z_{1}G_{11}^{-1}+G_{12}^{-1}\\ Z_{1}G_{21}^{-1}+G_{22}^{-1}\\ Z_{1}G_{31}^{-1}+G_{32}^{-1}\\ Z_{1}G_{41}^{-1}+G_{42}^{-1} \end{array}\right\}\kappa_{x}+\xi \left\{\begin{array}{c} \kappa_{x}\\ 0\\ 0 \end{array}\right\}$$

The nonsymmetrical wide flange I-beam stress can be obtained from Equation (14).

## 3. Finite Element Analysis Model

**Figure 3:**
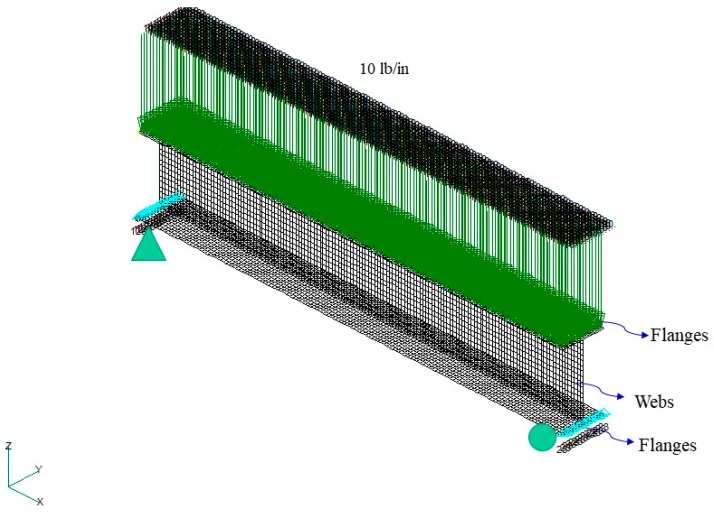
Geometric model and boundary conditions of I-beam.

**Figure 4:**
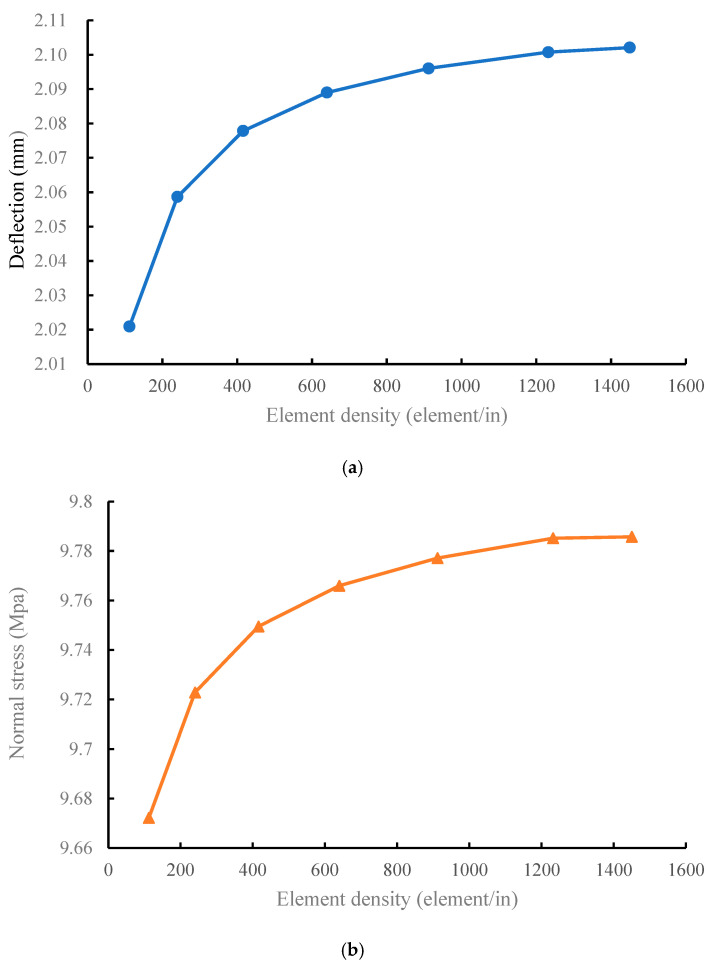
Convergence and divergence analysis of element number of I-beam. (**a**) Deflection, and (**b**) normal stress.

This study employed MSC NASTRAN numerical analysis software. The I-beam was divided into two parts, the web and flange, to construct its geometric model. The material used was AS4/3501-6 carbon/epoxy, and its properties were $E_{11}=18.4Msi$, $E_{22}=1.6Msi$, $\upsilon_{12}=0.28$, and $G_{12}=0.95Msi$. Laminates with material properties were set to 2D orthotropic. The beam length, flange width, and web height was 5, 0.5, and 0.75 in, respectively, and the web lamination was ${\left[90/45/-45\right]}_{s}$. The symmetric flange lamination was ${\left[90/45/-45/0/0\right]}_{s}$, and the nonsymmetric flange lamination was $\left[0_{4}/90/45/-45/-45/45/90\right]$. In terms of the boundary conditions, it was assumed that a simple support beam was applied to the upper flange with a uniformly distributed force (Figure 3). The element was set as a four-node quadrilateral element. In the element convergence analysis, when the element distribution density was >1200 elements/in, the stress and deflection of the beam tend to converge (Figure 4a,b). Therefore, the element density in the future will be maintained for at least 1200 elements/in to ensure the stability of the analyzed data.

## 4. Results and Discussion

**Figure 5:**
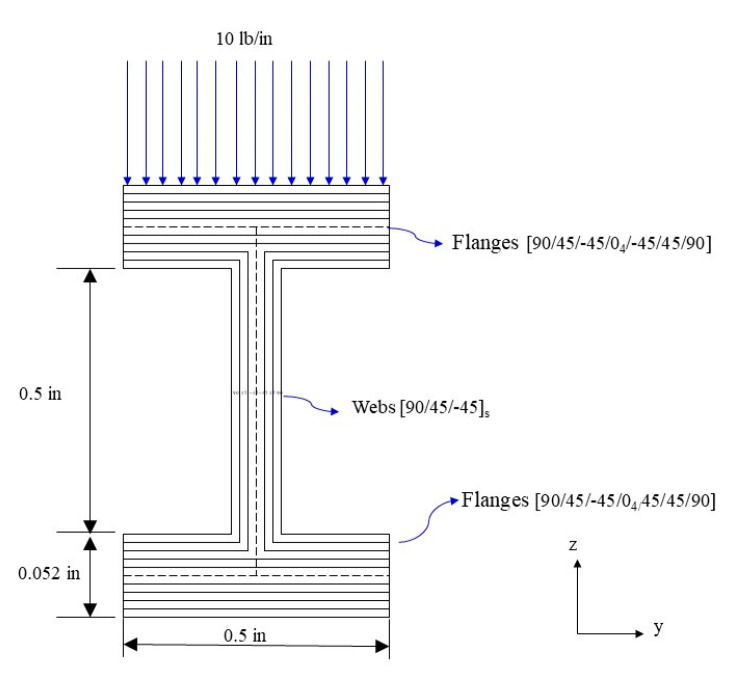
Laminated I-beam cross section.

The following section discusses a simple supported laminated I-beam made of AS4/3501-6 carbon/epoxy. The beam length, width, and height were 20, 0.50, and 0.75 in, respectively. The symmetric flange lamination was ${\left[90/45/-45/0/0\right]}_{s}$, the nonsymmetric flange lamination was $\left[0_{4}/90/45/-45/-45/45/90\right]$, the web lamination was ${\left[90/45/-45\right]}_{s}$, subject to a distributed load of 5 lb/in, and the flange and web lamination thicknesses were both 0.0052 in (Figure 5). In the FEM analysis, the beam was divided into 24,640 elements and 25,245 nodes.

**Figure 6:**
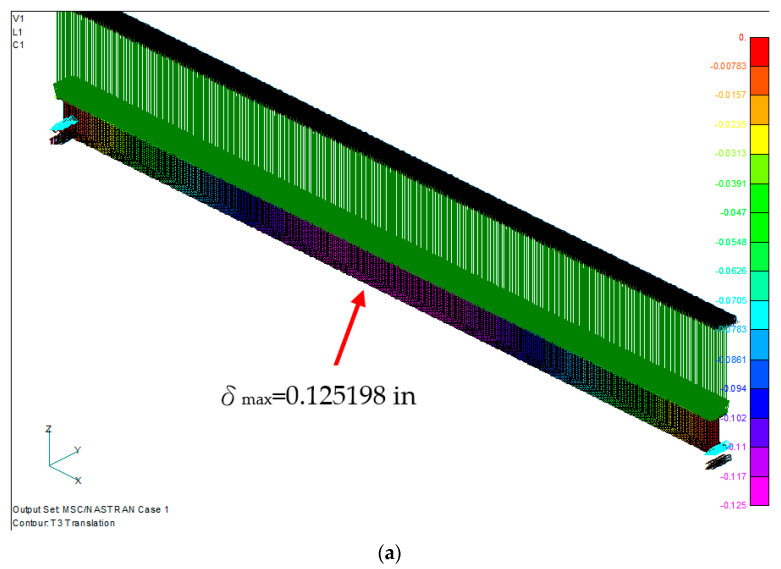

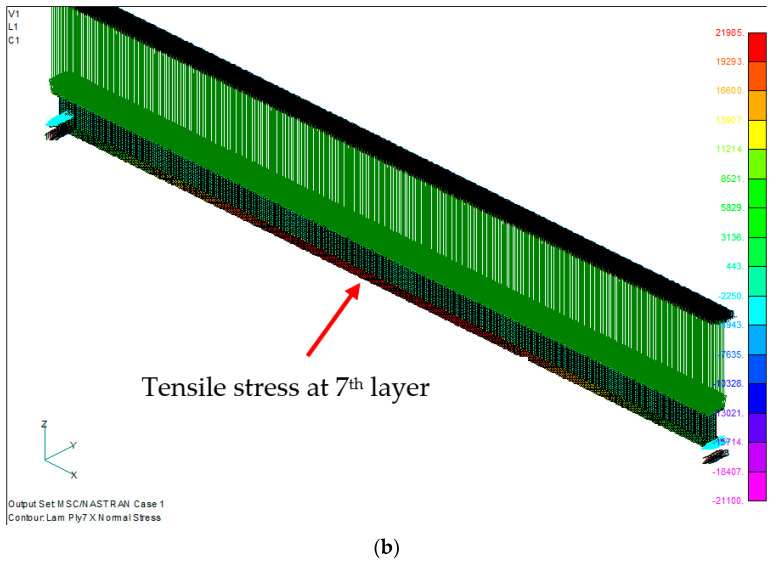
Symmetrical laminated I-beam. (**a**) Displacement distribution, and (**b**) maximum bending stress distribution at the 7th ply of the lower flange.

**Figure 7:**
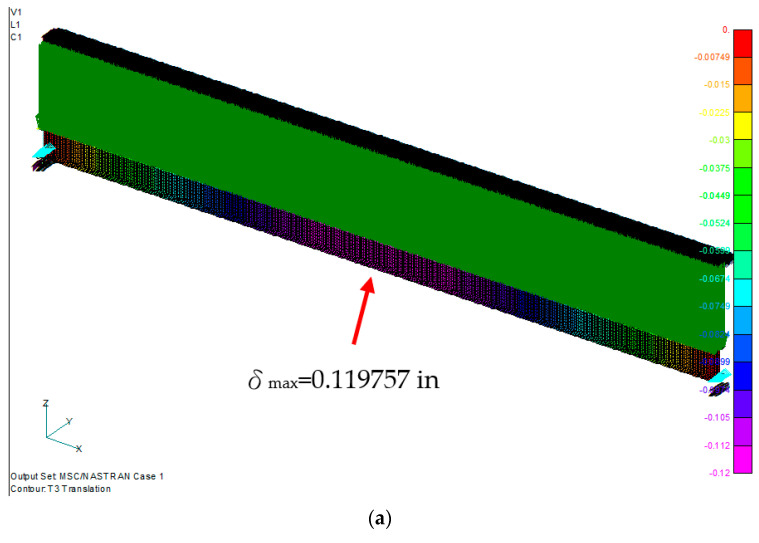

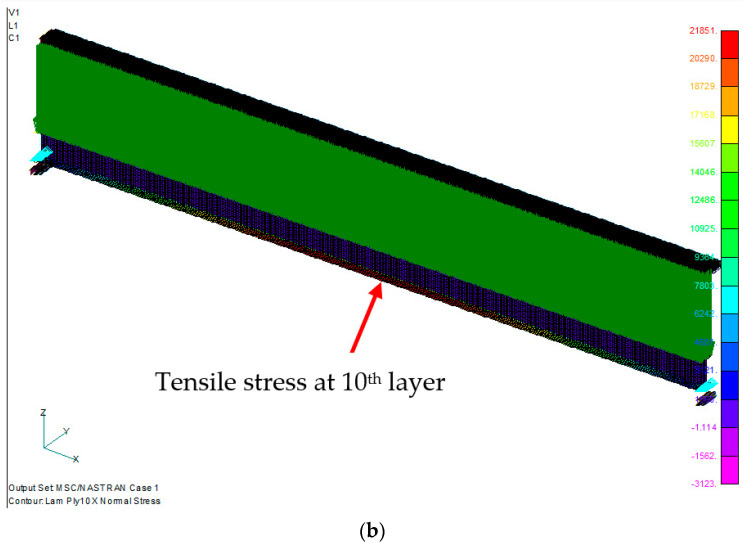
Nonsymmetrical laminated I-beam. (**a**) Displacement distribution, and (**b**) maximum bending stress distribution at the 10th layer of the lower flange.

The maximum deflection distribution of the symmetrical laminated I-beam was in the middle of the beam, with a value of 0.125198 in. The maximum stress is 21,985.48 psi at the 7th layer of the lower flange, which is under tensile stress (Figure 6). The maximum stress of the lower flange of the nonsymmetrical laminated I-beam shown in Figure 7 is 21,850.61 psi at the 10th layer of the lower flange. The maximum deflection position is in the middle of the beam, with a value of 0.119757 in.

Beam lengths of 25, 20, 15, 10, 7.5 and 5 in were used for the FEM analysis. The results are listed in Table 1 and Table 2. The deflection analysis results indicate that the symmetry is greater than the asymmetry. The nonsymmetrical maximum bending stress was greater than the symmetrical maximum bending stress. This is because the nonsymmetric laminate moved by 0° layers to the outermost flange layer. The 0° fiber layer was far from the centroid. Therefore, the overall rigidity can be increased by approximately 4–5% without changing the material. In addition, beams are discussed as beam or flatbed structures during the formulation derivation. The data indicate that the maximum normal stresses on the wide and narrow flanges are nearly identical. It can be deduced that the flange size does not affect the stress calculation data of the analytical solution.

**Figure 8:**
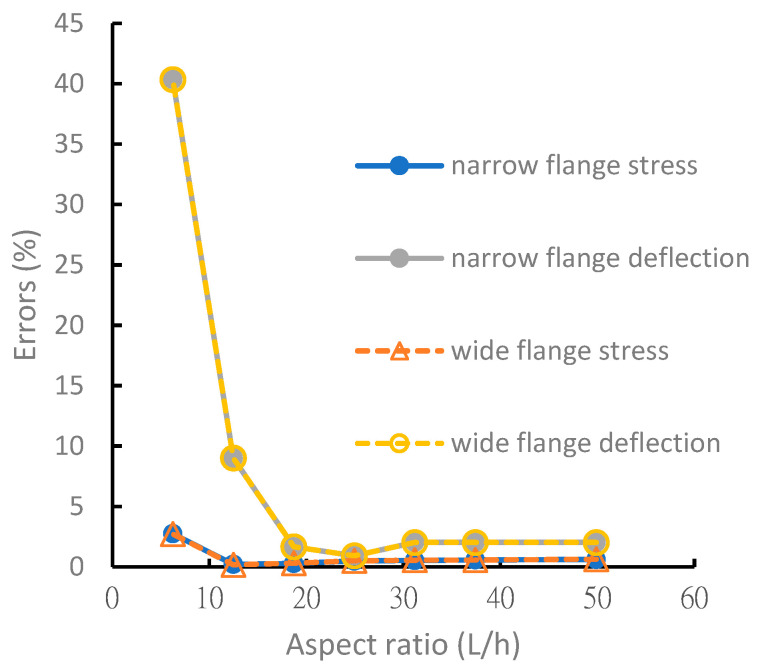
Analysis error comparison of symmetric laminated I-beams under different aspect ratios.

**Figure 9:**
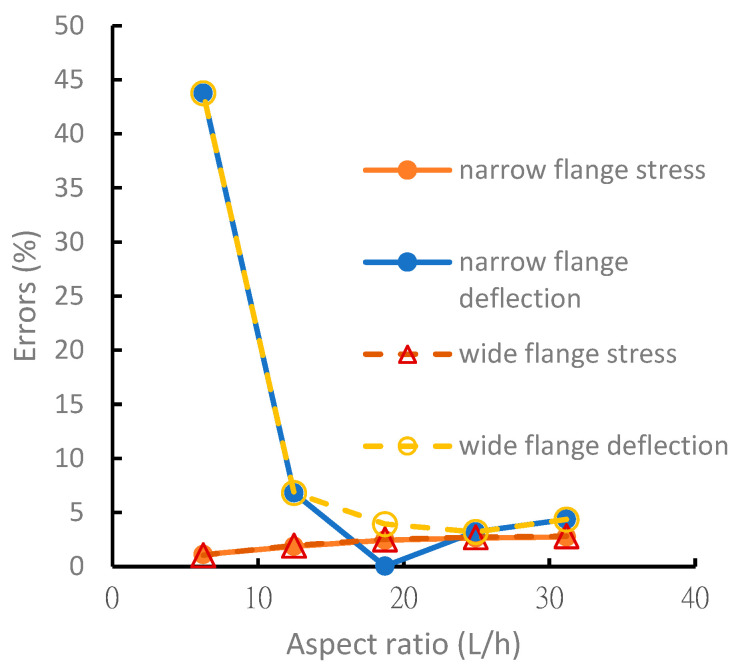
Analysis error comparison of nonsymmetric laminated I-beams under different aspect ratios.

As shown in Figure 8 and Figure 9, the analytical solutions for normal stresses on the symmetric and asymmetric beam flanges have excellent accuracy within 5% of the FEM solution. When the aspect ratio was >15, the analysis error of the deflection was within 5%. When the aspect ratio was <15, the error of the theoretical value increased because the theoretical analysis ignored the shear force. Beams with shorter lengths no longer possessed the properties of a beam structure, and the assumptions of the beam theory are not applicable to the analysis of short beams. The error was significant, particularly the deflection error. When the aspect ratio of the beam was <15, the error was >40%.

## 5. Conclusions

This study discusses the maximum deflection and bending stress of a simple support laminated composite I-beam under a distributed force, and compares the error between the theoretical and finite element solutions. The finite element analysis model constructed in this study can obtain better convergence data using element convergence analysis. From these discussions, the following conclusions were drawn.

(1) Analytical solutions provide a broad and rough assessment of composite beam structures. The scope of applicability of the formula was defined in detail by evaluating it in this study. Thus, the structural analysis of the composite beams can accurately obtain analysis data.
(2) The composite beam theory only considers plane strain, and there is a significant error in the strain and stress analyses of the short beam structure. When the aspect ratio of the beam was >15, the error in the analytical solution was <5%. Analytical solutions exhibited the best reliability for the normal force assessment of symmetric or nonsymmetric laminated beams. Therefore, the derived formula is suitable for thin and long beams.
(3) The change in the fiber angle of the laminate will improve the rigidity of the structure without changing the material. When the lamination sequence was changed and 0° ply was placed on the outermost layer of the flange, the effective stiffness of the nonsymmetric beam increased by 4–5% compared with that of the symmetric beam.

This study first discussed the accuracy of the analytical solution of the composite I-beam to understand the scope of the application of the basic theory. In future, planning will focus on basic structural beams, such as T-, L-, U-, and X-shaped composite beams. These structural beams are nonsymmetric geometric structures, and there is currently no relevant theoretical solution. Furthermore, for fatigue failure analysis of the laminated beam structures, the stress distribution of the holes on the beam and the failure behavior of the material after heating are all important topics to be discussed.

## Figures and Tables

**Table 1:** Comparison between theoretical and FEM solutions of symmetrical laminated I-beam.

	Max. Normal Stress (psi)			Max. Deflection (in)		
Length (in)	Theoretical Analysis Solution		FEM Solution	Theoretical Analysis Solution		FEM Solution
	Narrow Flange	Wide Flange		Narrow Flange	Wide Flange	
25	34,506.087	34,514.798	34,310.910	0.308363	0.308342	0.302105
20	22,083.896	22,089.471	21,978.570	0.126306	0.126297	0.125149
15	12,422.191	12,425.327	12,384.600	0.039964	0.039961	0.040620
10	5,520.974	5,522.368	5,531.170	0.007894	0.007894	0.008671
7.5	3,105.548	3,106.332	3,133.002	0.002500	0.002498	0.003072
5	1,380.243	1,380.592	1,419.221	0.000493	0.000493	0.000827

**Table 2:** Comparison between theoretical and FEM solutions of nonsymmetrical laminated I-beam.

	Max. Normal Stress (psi)			Max. Deflection (in)		
Length (in)	Theoretical Analysis Solution		FEM Solution	Theoretical Analysis Solution		FEM Solution
	Narrow Flange	Wide Flange		Narrow Flange	Wide Flange	
25	35,044.783	35,070.324	34,096.23	0.301742	0.30172	0.289010
20	22,428.661	22,445.007	21,843.75	0.123593	0.12359	0.119714
15	12,616.122	12,625.317	12,311.94	0.039106	0.03910	0.0388464
10	5,607.165	5,611.252	5,502.920	0.007725	0.00772	0.0082852
7.5	3,154.031	3,156.329	3,120.298	0.002444	0.00244	0.0029312
5	1,401.791	1,402.813	1,417.687	0.000483	0.00048	0.0008585
